# Transdiagnostic treatment of emotional disorders for women with multiple sclerosis: a randomized controlled trial

**DOI:** 10.1186/s12905-020-01109-z

**Published:** 2020-10-31

**Authors:** Nabi Nazari, Akram Aligholipour, Masoud Sadeghi

**Affiliations:** 1 Department of Psychology, Faculty of Human Sciences, Lorestan University, Khorramabad, Iran; 2 Department of Psychology, Islamic Azad University, Hamadan Branch, Hamadan, Iran

**Keywords:** Unified protocol, Emotion regulation, Comorbidity, Depression, Anxiety

## Abstract

**Background:**

Multiple sclerosis (MS) is a chronic, unpredictable, neurodegenerative disease, significantly associated with psychological, behavioral, cognitive, and emotional consequences. MS is more common in females than males and frequently affects women during their reproductive years. Despite the frequent mental disorders, comorbidities, and emotional problems in People with MS (PwMS), these conditions are too often underdiagnosed and undertreated.

**Objective:**

This study aimed to examine the efficacy of a group format of the Unified Protocol (UP) for the Transdiagnostic treatment of depression and anxiety disorders in females with MS.

**Methods:**

In the present study, Sixty-four adult females diagnosed with MS were randomized to either the UP (*n* = 32) or treatment-as-usual conditions. The assessment protocol included semi-structured clinical interviews and self-reports evaluating diagnostic criteria, depression, anxiety and worry symptoms, emotional regulation, and affectivity.

**Results:**

Repeated measure analysis of variance (ANOVA) revealed that the UP significantly improved depression scores [Cohen’s *d* = − 2.11, 95% CI (− 2.72, − 1.50)], anxiety scores [Cohen’s *d* = − 3.34, 95% CI (− 4.01, − 2.58)], positive and negative affect scale (PANAS)-positive affect scores [Cohen’s *d* = 1.46, 95% CI (1.46, 2.01)], PANAS-negative affect scores [Coen’s *d* = − 2.21, 95% CI (− 2.84, − 1.60)], difficulties emotion regulation scale scores [Cohen’s *d* = 1.40, 95% CI (− 0.87, − 0.03)], and Worry scale scores [Cohen’s *d* = − 0.45, 95% CI (− 0.95, − 0.04)] at the end of treatment relative to compared to the control condition. Also, treatment gains were maintained at the three-month follow-up (*p* < 0.001).

**Conclusion:**

The findings provide the support that the UP could be an additional efficient psychological treatment for females with MS.

*ISRCTN Number*: ISRCTN95459505.

## Background

Multiple sclerosis (MS) is a chronic, unpredictable, neurodegenerative disease of the central nervous system (CNS). MS is significantly associated with psychological, behavioral, cognitive, and emotional consequences [1]. Depression is a significant determinant of well-being [2] that affects approximately 75% of people with MS (PwMS) at some point during their disease [3, 4]. The lifetime prevalence of depression in females with Multiple Sclerosis is estimated about 50% [5]. Depression and anxiety can negatively impact on functioning, disability, physical impairment, pharmacological therapy adherence, and quality of life in PWMS [6, 7]. In addition to specific-disorder, Psychological comorbidity is common in PWMS [8] and is correlated with greater disability over time [9]. In addition to psychological consequences, PWMS have frequently reported emotional dysregulation. For example, 73% of PwMS endorsed subjective symptoms of emotion dysregulation such as irritability or crying during the last month. In this sense, emotional regulation mechanisms affected by MS is receiving more attention [10]. Progressive nerve demyelination, neuro-axonal loss, and axonal degeneration impair the ability to neural communication [11] frontal lobes, prefrontal cortex, and Amygdala [12]. These brain areas have critical roles in emotional regulation and psychosocial skills [13, 14]. Importantly, a high prevalence of depression associated with no treatment receive or anxiety disorders comorbidity has been associated with high suicide risk in PwMS [15]. Suicidal behaviors in PwMS are two times higher than the general population [16]. Like in the general population, women were at higher risk for attempted suicide compared to men, whilst men were at higher risk for completed suicide attempts [17]. Depression, maladaptive coping, and emotional dysregulation were the most potent predictors that have predictive accuracy for suicidal ideation as many as 85%. [18] in PwMS. Despite the frequent mental disorders, comorbidities, and emotional problems in PwMS, these conditions are too often underdiagnosed and undertreated [19].

### Current issues and treatment approaches

In a neurologic setting, evidence highlights the weakness of the DSM criteria application [20]. The heterogeneous nature of the MS syndrome and the potential for confusing specific somatic complaints of MS with depression symptoms may lead to falsely elevated underdiagnoses rates. Also, the efficacy of the diagnostic specific protocols is questioned with recent researches that demonstrate in the complicated cases and emotional disorders [21]. Cognitive Behavioral Therapy (CBT) programs have demonstrated effectiveness in promoting mental health in PwMS [22, 23]. However, disorder-specific interventions and treatments based on primary and secondary diagnoses are not suggested to be effective with complex cases [24]. Recent findings have shown that CBT was less efficient than other interventions in the psychological treatment of PwMS [25]. Transdiagnostic and integrated therapies have emerged as recommended approaches for the treatment of several co-occurring mental health disorders, as they provide a more parsimonious [26], and more efficient strategy to working with comorbid presentations [27]. Some studies have suggested that a transdiagnostic treatment approach for PwMS can be appropriate [28].

Transdiagnostic approaches refer to the identification of the etiology and maintenance mechanisms that are common in multiple disorders [29]. In the case of emotional disorders, neuroticism has been considered a key etiology mechanism shared by all emotional disorders [30]. Other mechanisms identified have been rumination, suppression, anxiety sensitivity, and misappraisal [31], which are frequently reported in PWMS [32]. These mechanisms can increase or maintain persistent negative emotions and may potentially affect physical as well as psychological functioning. From this perspective, transdiagnostic treatments consist of a set of techniques which are served to target an identified set of underlying core processes [29]. Emotion regulation seems to play a critical role in the treatment of complex cases, diagnoses with a combination of psychological risk factors, or comorbidities [33]. There is evidence that supports the application of emotion regulation in promoting adaptive emotion regulation among women with mental disorders [34]. Women with MS experience higher rates of negative emotions related to different situations such as support family members, body image, pregnancy worry, uncertainty about the relationship, and sexual dysfunction [35]. Typically, MS women exhibit emotion-focused coping styles more often than males [36]. Epidemiological data also highlight a rapid increase in the female: male ratio of MS [37]. Collectively, the application of an emotion-focused treatment for MS women could be beneficial.

The Unified Protocol (UP) is a CBT transdiagnostic emotion-focused therapy [38, 39]. The UP has been manualized to be applied to the treatment of anxiety disorders, depression, and other emotional disorders in which emotion dysregulation is a core component [40]. The protocol has been adopted in 12–14 sessions in a group format [41]. Numerous studies have supported the efficacy of the UP in improvements on anxiety and depression symptoms, functional impairment and wellbeing [39, 42], chronic diseases [43], and social, job, and general performance [44].

### Current study

Iran has one of the highest MS prevalence and incidence in the world with a high MS sex-biased ratio (female to male ratio 3.47:1). The MS incidence in Iranian females is 44/100,000 95% CI: (36–62) [37]. Studies have identified genetic and hormonal sex and gender differences in MS [45, 46]. New studies demonstrated gender influences on the frequency of anxiety [47]. This single-sex study aimed to examine the efficacy of a group format of the UP for depression or anxiety symptoms in adult MS women with difficulties in emotion regulation. We hypothesized that MS women who participate in the UP intervention group would demonstrate significant improvements in emotion regulation, affectivity, depressive, and anxiety and worry outcomes at post-treatment and 3-month follow-up, compared with the treatment-as-usual (TAU) group.

## Methods

### Participants

**Fig. 1:**
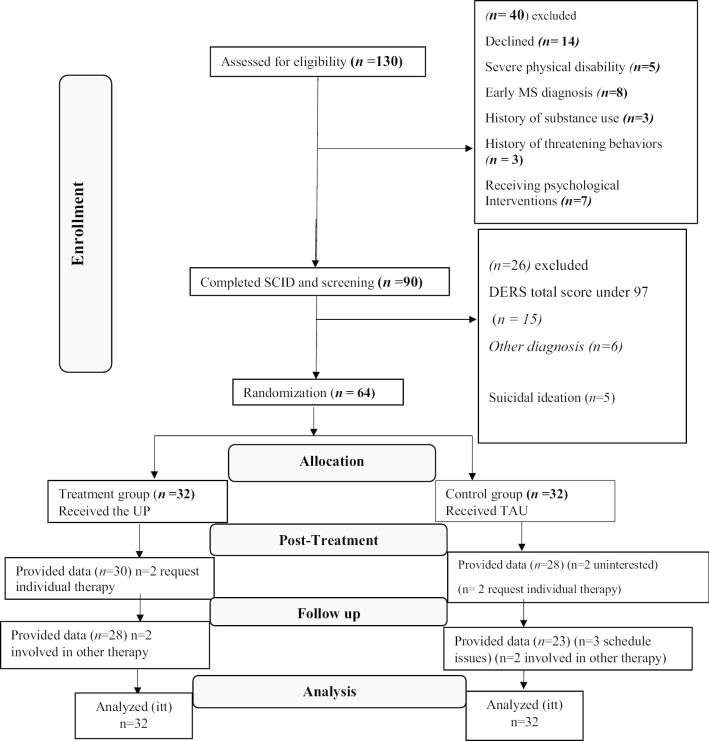
The participants flow chart diagram

The consort diagram is illustrated in Fig. 1. Forty of 130 individuals assessed for the initial eligibility were excluded. Of these, fourteen patients declined to participate or being unwilling to risk possible randomization to the control group. After a medical evaluation, Ninety potential participants were invited to an assessment, including a face-to-face semi-structured clinical diagnostic interview. At this step, fifty patients did not meet a principal diagnosis for depression and anxiety disorders, neither comorbidity associated with emotion regulation deficits or difficulties in emotion regulation. Four patients were identified with a suicidal ideation or behavior risk factors and referred to psychiatric intervention. A total of 64 women who obtained a signed written consent form (mean age = 35.13 years, *SD* = *5.28*) were selected for randomization. Baseline assessment is demonstrated in Table 1.

**Table 1:** Demographic characteristics of the sample (N = 64)

Item (N = 64)	Value	Test	*P*
*Categorical variables*			
MS duration*, n* (%)			
3–6	44 (68.8)	χ^2^ = 9.00	0.003
6 and higher	20 (31.2)		
Marital status*, n* (%)			
Single	25 (39.1)	χ^2^ = 3.29	0.07
In relationship	39 (60.9)		
Education *n* (%)			
Primary education	11 (17.2)		
Bachelor	30 (46.9)	χ^2^ = 8.65	0.01
Master+	23 (35.9)		
SCID-I–IV findings *n* (%)			
Depressive disorder	29 (45.3)	χ^2^ = 0.56	0.45
Anxiety disorder	35 (54.7)		
Continues variables *M (SD)*			
Age (year)	35.13 (5.28)	*t*(62) = 0.50	0.61
MS duration (year)	4.80 (1.37)	*t*(62) = 1.03	0.30
PANAS-PA	26.00 (3.75)	*t*(62) = 1.55	0.12
PANAS-NA	27.42 (2.72)	*t*(62) = − 0.6	0.15
PSWQ	47.55 (9.46)	*t*(62) = − 1.23	0.22
HADS-A	12.6 (1.38)	*t*(62) = − 1.89	0.06
HADS-D	12.84 (1.63)	*t*(62) = − 1.24	0.21
DERS	129.03 (15.54)	*t*(62) = − 1.5	0.13

*n* frequency, *y* years, *M* mean, *SD* standard deviation, *PANAS-PA* Positive and Negative Affect Schedule-Positive Affect, *PANAS-NA* Positive and Negative Affect Schedule-Negative Affect, *HADS-A* The hospital anxiety and depression scale-Anxiety, *HADS-D* The hospital anxiety and depression scale-Depression, *PSWQ* Penn State Worry Questionnaire, *DERS* Difficulties in Emotion Regulation Scale, *SCID-I–IV* Structured Clinical Interview for DSM-IV Axis I Disorders

### Eligibility criteria

*Inclusion criteria included*: (a) fluent in Persian (b) at least 18 years of age, (c) a diagnosis of MS for three years or more, (d) received a diagnosis of depression or anxiety disorders (f) high score in difficulties emotion regulation scale (g) medical agreement or valid referral document for participation.

*Exclusion criteria included*: (a) present or history diagnosis of schizophrenia, psychosis, or organic mental disorder, (b) other chronic physical illnesses (e.g., cancer, diabetes) (c) pregnancy or Breast-feeding, (d) risk or history of threatening behaviors, (e) missed three consecutive sessions (f) receiving psychological interventions during one last year.

### Measures

*Structured Clinical Interview for DSM-IV Axis I Disorders* [48, 49] (SCID I–IV) is a structured clinical interview designed to assess Axis I diagnoses in psychiatric population studies. The reliability and validity of the procedure is well-established. The diagnostic agreement for most of the specific and general diagnosis was moderate to good (Kappa coefficient higher than 0.6). The interviewers reported the desirable implementation of the Persian version of SCID-I. Kappa was higher than 0.4 for all the diagnoses except for Generalized Anxiety Disorders. The Kappa was above 0.85 in most of the diagnoses, and in half, it was above 0.9, indicating acceptable reliability [50].

### Primary outcomes measures

*The hospital anxiety and depression scale* (HADS). The HADS [51] is a highly reliable screening measure for assessing anxiety and depression in PwMS. The HADS consists of 14-items, two sub-scales 7-items for anxiety (HADS-A), and seven items for depression (HADS-D). A suggested cutoff score of 11 demonstrated high sensitivity (90%) and specificity (92%) for the Anxiety subscale and high sensitivity (77%) and specificity (81%) for the Depression subscale [52]. This scale demonstrated acceptable reliability in this study (*α* = 0.90).

*Difficulties in Emotion Regulation Scale* (DERS). The DERS [53] is a 36-item, self-report questionnaire that measures overall difficulties in emotion regulation. The DERS consists of six subscale: (1) no acceptance of emotional responses, (2) difficulties engaging in goal-directed behavior, (3) impulse control difficulties, (4) the lack of emotional awareness, (5) limited access, and (6) lack of emotional clarity. Respondents rated their emotional state on 1 (almost never) to 5 (almost always). The total score range of 36–180. A recent study has found that a DERS total score above 97 identified a clinical sample [54]. DERS has high internal consistency (α = 0.93). Internal consistency in the current study was acceptable (*α* = 0. 92).

### Secondary outcomes

*The Positive and Negative Affect Schedule* (PANAS). The PANAS [55] is a brief self-report scale that determines dimensions of positive and negative affect with two independent ten descriptors [55]. The PANAS demonstrates the two core dimensions of mood positive affect (PA) and negative mood affect (NA). Each item is rated on a five-point scale with a range from very slightly (1) to extremely (5), indicating the extent that the participant has experienced that feeling over the past month. The PANAS has shown highly internally consistent, largely uncorrelated PA (0.89) to NA (0.95), whereas the discriminant correlations are quite low [55]. Internal consistency in the current study was acceptable (*α* = 0.85).

*Penn State Worry Questionnaire (*PSWQ*).* The PSWQ [56] is a 16-item self-report measure that determines an individual's tendency to worry as well as intensity and excessiveness of worry on a scale of 1 (not at all typical of me) to 5 (very typical of me). The PSWQ has demonstrated reliable psychometric properties, suitable internal consistency, and test–retest reliability in the local MS population. This measure is suggested for transdiagnostic approach assessments. Internal consistency in the current study was acceptable (α = 0.83*).*

### Procedure

The study was a single-blind, single-sex, parallel randomized controlled trial comparing psychological intervention group, based on the UP, with a TAU control group. The study, including all assessments and treatments, was conducted at the MS Clinic, located within the MS Centre at Sina hospital. All methods and procedures for the study were reviewed and approved by the Institutional Human Research Ethics Committee and the National Institute for Medical Research and Development before the first participant enrollment, prospectively. Participant recruitment efforts included notifying MS clinics, and MS associations through the use of brochures and posters. The schedule of the intervention demonstrated in Table 2.

**Table 2:** Study schedule of enrolment, intervention, and assessment

Assessment	Enrollment schedule	Intervention schedule	Follow-up schedule
Duration	2 months	14 weeks	3 months
Informed consent	×		
SCID-I–IV interview	×	×	×
Demographic question	×		
Intervention			
Unified protocol		$\longleftrightarrow$	
Treatment as usual		$\longleftrightarrow$	
Primary outcome			
HADS	×	×	×
DERS	×	×	×
Secondary outcome			
PANAS	×	×	×
PSWQ	×	×	×

*SCID-I–IV* Structured Clinical Interview for DSM-IV Axis I Disorders, *PANAS* Positive and Negative Affect Schedule, *HADS* The hospital anxiety and depression scale-Depression, *PSWQ* Penn State Worry Questionnaire, *DERS* Difficulties in Emotion Regulation Scale

First, interested women, who contacted, were notified about the goals of the study, benefits (e.g., improving health outcomes), and risks (e.g., the risks involved in this study are not greater than everyday life), session numbers, randomization, and group allocation chance. Only women who agree to participate in the study were requested to present their physician agreement or referral to the study participation. The neurologists, non-informed about the study, evaluated physician agreements, referrals, medical documents, and examined the subjects. After the eligibility criteria related to medical conditions obtained; the participants completed the assessment protocol. Individuals who met the SCID-I–IV criteria for depression or anxiety disorders were requested to endorse the self-report measures. At last, only consented subjects who received a diagnosis of depression or anxiety disorders in SCID and obtained a high self-report score in DERS > 97, the HADS-A score ≥ 11, and the HADS-D score ≥ 11, were selected for randomization. The participants were informed that they could withdraw the consent or stop participating, any point of the study. Also, they were free to skip specific questions and continue participating. The outcomes were assessed at three time-points: Time 1: pretreatment to pre-allocation include baseline, Time 2: immediate after intervention: post-treatment assessment, and Time 3: immediate at the end of the three-month follow-up.

### Sample size

The Sample size for a repeated measure analysis of variance (ANOVA) with two groups: UP vs. TAU), and three measurement times (baseline, post-treatment, and three-month follow-up) conducted using GPower version 3 analysis [57]. A priori power analysis was conducted, using an alpha of 0.05, a power of 0.95, effect size (*f* = 0.3), and 0.3 correlation among repeated measures to determine the sample size required. According to GPower, the desired total sample size was 52. Therefore, 64 participants were recruited, allowing for a 15% loss of data.

### Randomization and blinding procedures

Randomization was performed using the concealed computerized Permuted block randomization method. The block size was 4. The concealed was disclosed at the end of study. An independent statistician carried out the randomization and informed the patient and researcher about the allocation. To masking condition assisting, participants were instructed not to disclose any information about the intervention and pervious diagnostic status. Psychological evaluators, assessors, and statistic investigators were blinded to the intervention, participants' group, and pervious diagnostic status.

### Interventions

#### The unified protocol intervention

The program and sessions were structured based on the latest comprehensive published manual developed by Barlow and colleagues [38, 39]. Group therapy consists of 14 weekly two-hour sessions. The treatment content is included topics about Motivation, psychoeducation, mindfulness, cognitive flexibility, emotion-driven behavior, and emotional avoidance, interoceptive exposure (IE), in vivo exposure, and relapse prevention. The summary of each module content and intervention schedule is demonstrated in Table 3. (See Additional file 1 for the more detailed description.)

**Table 3:** Content and the number of sessions for module

Module	Content and the number of sessions for module
One	Setting goals and maintaining motivation (1 session)
Two	Understanding emotions (1 sessions)
Three	Mindful emotion awareness (2 sessions)
Four	Cognitive flexibility (2 sessions)
Five	Countering emotional behaviors (1 sessions)
Six	Understanding and confronting physical sensations (1 session)
Seven	Emotion exposures (5 sessions)
Eight	Recognizing accomplishments and looking to the future (1 session)

#### Treatment-as-usual intervention

The control group received the TAU that consists of 14 weekly two hour sessions. The program included psychoeducation (2 sessions), relaxation and breathing training (1 session), sharing experiences (4 sessions), life-long MS considerations (4 sessions), marital and parental counseling (2 sessions), and lifestyle consideration in MS (1session). This treatment could be considered as a psychosocial skill or social support intervention delivered in routine care focused on reducing negative emotions.

### Risk

Routine medical and psychological evaluations were accomplished before all activities (e.g., assessments, interviews, and treatment sessions). Regarding safety, the medical health care staff included two physicians and two experienced nurses also alerted in case of emergency conditions during all activities.

### Data analysis

All analyses were performed using SPSS version 25, (SPSS Inc., Chicago, IL), with a two-sided 5% level of significance, following an Intention-to-Treat (ITT) analysis approach. Given that the analysis was based on ITT principles, the data for all randomized 64 individuals were included in the final report. To missing data handle, the last observation-carried-forward (LOCF) method was considered as a next point for dropping data. The data are presented as the mean and standard deviation for continuous variables and numbers or percentages for categorical variables. An independent *t*-test was conducted to explore whether the participants were equivalent at baseline (Time 1). Repeated measure ANOVA 2 (Treatment: UP vs. TAU) × 3 (times: baseline, post-treatment, and three-month follow-up) was used to investigate treatment differences and to identify within-group differences. The Cohen's *d* effect sizes were calculated using the difference between the UP means, and the TAU means divided by a standard deviation for the data.

## Results

### Descriptive characteristics at baseline

The demographic characteristics of the sample were illustrated in Table 1 (see Table 1). The mean and *SD* age of the participants in the UP was (*M* = 33.49 years, *SD* = 4.67) with MS duration range from 3 to 6.67 years and of control group was (*M* = 34.10 years, *SD* = 5.01) with MS duration range from 3 to 6.25 years. Two women from the UP group left the experiment before Time 2. On average, participants had a very high degree of adherence and protocol well tolerated; 90% (*n* = 28) of the UP group completed all the treatment sessions and completed all the measures at post-treatment and follow up. Four women from the TAU group did not complete the post-treatment, and seven participants dropped out at the end of follow up. At Time 3, 78% (*n* = 51) participants completed the study.

### Treatment results

Repeated measure ANOVA was conducted on HADS-D. The results showed a significant main effect for group, *F*(1, 62) = 116.55, *p* < 0.001, η^2^*p* = 0.65. Between groups analyses showed that the UP participants obtained statistically significant less HADS-D scores than TAU at post-treatment [*t*(1,62) = 9.94, *p* < 0.001, Cohen’s *d* = − 2.11 95% CI (− 2.72, − 1.50)]. Also, there was a significant group × time interaction, *F*(2, 124) = 64.63, *p* < 0.001, *η*^*2*^*p* = 0.51.

Repeated measure ANOVA was conducted on HADS-A. The results showed a significant main effect for group, *F*(1, 62) = 158.23, *p* < 0.001, η^2^*p* = 0.72. Between groups analyses showed that the UP participants obtained statistically significant less HADS-A scores than TAU at post-treatment [*t*(1,62) = 12.92, *p* < 0.001, Cohen’s *d* = − 3.34, 95% CI (− 4.01, − 2.58)]. Also, there was a significant group × time interaction, *F*(2, 124) = 63.27, *p* < 0.001, *η*^*2*^*p* = 0.50.

Repeated measure ANOVA was conducted on DERS. The results showed a significant main effect for group, *F*(1, 62) = 36.46, *p* < 0.001, η^2^*p* = 0.37. Between groups analyses showed that the UP participants obtained statistically significant less DERS scores than TAU at post-treatment [*t*(1,62) = , *p* < 0.001, Cohen’s *d* = 95% CI (,)]. Also, there was a significant group × time interaction, *F*(2, 124) = 22.02, *p* < 0.001, *η*^*2*^*p* = 0.26.

Repeated measure ANOVA was conducted on PANAS-PA. The results showed a significant main effect for group, *F*(1, 62) = 37.68, *p* < 0.001, η^2^*p* = 0.38. Between groups analyses showed that the UP participants obtained statistically significant less PANAS-PA scores than TAU at post-treatment [*t*(1,62) = 5.83, *p* < 0.001, Cohen’s *d* = 1.46, 95% CI (1.46, 2.01)]. Also, there was a significant group × time interaction, *F*(2, 124) = 27.48, *p* < 0.001, *η*^*2*^*p* = 0.31.

Repeated measure ANOVA was conducted on PANAS-NA. The results showed a significant main effect for group, *F*(1, 62) = 156.25, *p* < 0.001, η^2^*p* = 0.59. Between groups analyses showed that the UP participants obtained statistically significant less PANAS-NA scores than TAU at post-treatment [*t*(1,62) = , *p* < 0.001, Cohen’s *d* = − 2.21, 95% CI (− 2.84, − 1.60)]. Also, there was a significant group × time interaction, *F*(2, 124) = 161.23, *p* < 0.001, *η*^*2*^*p* = 0.62.

Repeated measure ANOVA was conducted on PSWQ. The results showed a significant main effect for group, *F*(1, 62) = 24.90, *p* < 0.001, η^2^*p* = 0.29, and a significant main time effect. Between groups analyses showed that the UP participants obtained statistically significant less PSWQ scores than TAU at post-treatment [*t*(1,62) = , *p* < 0.001, Cohen’s *d* = − 0.45, 95% CI (− 0.95, − 0.04)]. Also, there was a significant group × time interaction, *F*(2, 124) = 19.24, *p* < 0.001, *η*^*2*^*p* = 0.24 (Table 4).

**Table 4:** Mean and SD in baseline, post-treatment and follow up

Measure	Control group (TAU) *n* = *32*			Intervention group (UP) *n* = *32*		
	Time 1	Time 2	Time 3	Time 1	Time 2	Time 3
	M (SD)	M (SD)	M (SD)	M (SD)	M (SD)	M (SD)
PANAS-PA	26.72 (3.97)	26.63 (3.74)	27.03 (4.22)	25.28 (3.44)	31.97 (3.59)	34.43 (3.71)
PANAS-NA	27.22 (2.88)	28.06 (2.91)	28.22 (2.67)	27.63 (2.55)	22.00 (2.54)	23.34 (3.22)
PSWQ	46.1 (9.39)	46.50 (8.10)	44.37 (8.49)	49.0 (9.45)	41.38 (9.25)	43.4 (11.18)
HADS-A	12.47 (1.52)	12.06 (1.26)	11.78 (1.66)	13.1 (1.51)	7.25 (1.60)	7.34 (1.78)
HADS-D	12.4 (1.81)	12.90 (1.74)	12.37 (1.80)	12.9 (1.80)	8.12 (2.40)	6.94 (1.76)
DERS	125.1 (17.4)	120.0 (17.43)	125.1 (14.08)	133.0 (15.56)	98.1 (21.94)	93.9 (19.04)

The SCID-I–IV demonstrated 22 of 30 patients in the UP group (73.3%) no longer met diagnostic criteria for their principal diagnosis at the end of the study at Time 3. The SCID-I–IV demonstrated no worse condition for all participants at Time2 and Time 3.

## Discussion

MS is associated with a broad array of emotional disorders, negative symptoms, social interference, and physical disability that compromise well-being [4]. This study aimed to examine the efficacy of a group format of the UP for the transdiagnostic treatment of emotional disorders and symptoms in adult MS women with emotion dysregulation. The results indicated the UP effectiveness on changes in depression and anxiety symptoms and improvement of the emotion regulation at post-treatment. Also, treatment gains were maintained at the three-month follow-up.

Our findings revealed significant changes in depression measure, in anxiety measure, and in worry at 3-month follow up in the UP group. The results are consistent with studies that indicate the UP is effective in improving emotional disorders. In anxiety disorders, worrying is a critical maladaptive cognitive process contributing to the maintenance of the disorder, and worrying can be effectively targeted by promoting adaptive emotion regulation strategies.

Findings revealed significant changes in DERS at post-treatment regarding with TAU group. This study develops the UP benefits on difficulties emotion regulation scale which can potentially improve other clinical outcomes (e.g., anxiety symptoms) [58]. Also, the results provide supports the application of emotion regulation in promoting adaptive emotion regulation among women with mental disorders [34]. The improvement of emotion regulation can be associated with an improvement in depression and anxiety symptoms [59]. Furthermore, in line with our investigation, numerous researches have replicated the emotional regulation implication in the treatment of depression [60], anxiety disorders [61], excessive worry and psychological stress [31].

A large Cohen's *d* in the negative and positive affect was found with a higher significant effect on negative affect than positive affect. These results are the same that previous RCT, applying UP in emotional disorders samples that have found changes in neuroticism/negative affect after UP intervention [62]. Some studies have also found differences in extraversion/positive affect [63]. The reduction in neuroticism scores confirm the theory about the UP, an emotion regulation intervention targeting specifically neuroticism/negative affect [30], a psychopathology mechanism associated with the etiology of the emotional disorders [64]. Conceptually, the UP in a group-format may offer several benefits over the individual format such as in-session exposure in a group, validation, and support,

The current study could develop the UP as a transdiagnostic approach, consisting of five core modules and practical techniques for addressing different aspects of emotion regulation. Emotion dysregulation predicts quality of life, independently of disease severity and cognitive functioning [65]. Moreover, Emotional distress associated with maladaptive coping strategies is led to poor wellbeing rather than disease duration or severity [66] for example, emotional problems among mothers with MS negatively associate with the mother’s ability to cope with the disease and positively associate with depressive symptoms in their healthy partners [67]. Psychoeducational courses, emotional skills, and stress self-management techniques can be beneficial to enhance wellbeing in MS [68]. Awareness of the role of thoughts, beliefs, and their interaction facilitate coping in PwMS [69]. Interoceptive exposure is another component of the UP. In PwMS, bodily sensations are usually associated with high anxiety. interoceptive exposure and mindfulness may be beneficial and facilitate a controlled coping behavior, and less stress react, gradually [70]. PwMS focus on the disease consequences, which may be concluded to catastrophizing future, over-estimate threat and under-estimate their abilities to cope. Present-Focused Emotion, another core module in the UP, helps the patients to recognize their thoughts and feelings and concentrate on the current condition demands, which could make emotional experiences feel more under control and manageable.

The study revealed surprising findings. The participants who received an anxiety disorder diagnosis, 54.7% (*n* = 35) based on the SCID-I interview, were more than those who received a depressive diagnosis at baseline 45.3% (*n* = 29). This finding is contrary to current insight and epidemiologic data in PwMS [5]. This finding is critical because anxiety receives far less attention in MS. DERS scores are related to both depression and anxiety levels in the MS sample [10]. According to DERS mean score at baseline, difficulties with emotion regulation is very high in women patients. So, emotion-focused or skill-based interventions can be considered as an additional treatment in MS setting. Also, all women with depression or anxiety symptoms diagnosis in SCID-I–IV received a clinical HADS cutoff score or above. To diagnosis depression or anxiety symptoms in our sample with emotion dysregulation, the HADS generated evidence the same as SCID-I–IV. This recent finding can be considered as a cost-effective strategy in the future same sample trials.

We investigated the feasibility of the UP in a group format to an MS transdiagnostic sample with emotion regulation problems. According to evidence considering that emotion dysregulation is connected with less willingness to participate in psychological trials [71], we classified the sample as challenging to treat. According to the treatment retention rate in this study (79%), the treatment well tolerated. Also, the results are in line with the data provided previous trials [41, 72] confirm a significant improvement of patients treated in a group format, findings that are maintained in the follow-up.

The results from this trial should be interpreted in the context of several limitations. First, the single-sex design diminished the results' generalizability. Next limitation, the participants were generally well-educated, which can be enhanced their abilities to gain more the UP. That said, the group-format has some limitations. Attending to a group of patients in 90-min sessions meant spending less time on each individual patient. One strength point of this study was the SCID-I–IV application associated with the HADS screening at enrollment. Another strength point was an enrollment diagnostic based on comorbidity. The recent investigation is critical because the efficacy of a single protocol in the improvement of comorbid disorders in a sample of women with MS is a promising finding for clinicians and patients.

## Conclusion

Overall the findings provide support that the UP could be an additional efficient as a parsimonious; transdiagnostic treatment of emotional disorders for adult MS women. Developing and applying a single effective therapeutic protocol in diagnostic categories to target the main features of emotional disorders can be a cost-effective alternative in addition to their benefits in psychologist’s training and CBT dissemination compared to existing therapeutic protocols for specific clinical diagnoses [73].

## Supplementary information

**Additional file 1.** Consort.

## Data Availability

The data that support the findings of this study are available on request from the corresponding author.

## Abbreviations

ANOVA: Analysis of variance
CBT: Cognitive behavioral therapy
CNS: Central nervous system
DERS: Difficulties in emotion regulation scale
DSM: Diagnostic and statistical manual of mental disorders
HADS: The hospital anxiety and depression scale
IE: Interoceptive exposure
ITT: Intention-to-treat
LOCF: Last observation-carried-forward
MS: Multiple sclerosis
NA: Negative affect
PANAS: Positive and negative affect scale
PA: Positive affect
PSWQ: Penn state worry questionnaire
PwMS: People with MS
SCID I–IV: Structured clinical interview for DSM-IV axis I disorders
TAU: Treatment-as-usual
UP: Unified protocol
n: Frequency
y: Years
M: Mean
SD: Standard deviation
